# Towards a framework for agent-based image analysis of remote-sensing data

**DOI:** 10.1080/19479832.2015.1015459

**Published:** 2015-03-30

**Authors:** Peter Hofmann, Paul Lettmayer, Thomas Blaschke, Mariana Belgiu, Stefan Wegenkittl, Roland Graf, Thomas Josef Lampoltshammer, Vera Andrejchenko

**Affiliations:** ^a^ Interfaculty Department of Geoinformatics – Z_GIS, Salzburg University, Schillerstr. 30, Salzburg5020, Austria; ^b^ Department of Information Technology & Systems Management, Salzburg University of Applied Sciences, Salzburg, Austria

**Keywords:** agent-based image analysis, agent-based systems, remote sensing, object-based image analysis, autonomous systems, automation of image analysis

## Abstract

Object-based image analysis (OBIA) as a paradigm for analysing remotely sensed image data has in many cases led to spatially and thematically improved classification results in comparison to pixel-based approaches. Nevertheless, robust and transferable object-based solutions for automated image analysis capable of analysing sets of images or even large image archives without any human interaction are still rare. A major reason for this lack of robustness and transferability is the high complexity of image contents: Especially in very high resolution (VHR) remote-sensing data with varying imaging conditions or sensor characteristics, the variability of the objects’ properties in these varying images is hardly predictable. The work described in this article builds on so-called rule sets. While earlier work has demonstrated that OBIA rule sets bear a high potential of transferability, they need to be adapted manually, or classification results need to be adjusted manually in a post-processing step. In order to automate these adaptation and adjustment procedures, we investigate the coupling, extension and integration of OBIA with the agent-based paradigm, which is exhaustively investigated in software engineering. The aims of such integration are (a) autonomously adapting rule sets and (b) image objects that can adopt and adjust themselves according to different imaging conditions and sensor characteristics. This article focuses on self-adapting image objects and therefore introduces a framework for agent-based image analysis (ABIA).

## 1. Introduction

Since the beginning of the millennium, two major new technologies have influenced the remote-sensing community: the availability of very high resolution (VHR) remote-sensing data and object-based image analysis (OBIA). Although OBIA builds on several older concepts and methods such as image segmentation, the particular combination of these concepts allow applying multi-scale concepts (Burnett and Blaschke 2003). Both developments mentioned have led to a paradigm change in analysing remote-sensing data: from pixel-based to object-based methods (Blaschke *et al.* 2014). A widespread assumption is that the latter allows for the analysis of remote-sensing data beyond spectral statistical parameters, using further object properties such as shape and spatial context (Benz *et al.* 2004, Blaschke 2010). However – while invoking Blaschke and Strobl (2001) – one may first need to ask ‘What’s wrong with pixels?’ Instead of a comprehensive answer, we may refer to the rapidly growing body of literature where a significant number of authors identified an increasing dissatisfaction with pixel-by-pixel image analysis. Although this critique is not new (Cracknell 1998), see also Blaschke and Strobl (2001), Blaschke (2010) and Blaschke *et al.* (2014) for a more thorough discussion; these authors described a need for applications ‘beyond pixels’ and for specific methods and methodologies that support this.

This ready availability of high-resolution multi-band imagery coincided with the increasing awareness in remote-sensing literature that novel methods to extract meaningful and more accurate results were crucial. Likewise, what is fundamentally required in complex image processing tasks is a kind of ‘intelligence’. Here, the authors are very careful about venturing into artificial intelligence. Rather, what is needed is a kind of ‘geo-intelligence’ as described in Hay and Blaschke (2010).

In the first years of OBIA, segmentation was regarded to be inextricably linked to this concept. Indeed, segmentation provides the building blocks of OBIA (Hay and Castilla 2008, Lang 2008). Segments are regions that are generated by one or more criteria of homogeneity in one or more dimension (of a feature space). Thus, segments have additional spectral information compared to single pixels. It is, in principle, based on the spectral statistics of the segments’ underlying pixels. However, of even greater advantage than spectral per-object statistics is the additional spatial information for objects (Benz *et al.* 2004, Hay and Castilla 2008). It has been frequently claimed that this spatial dimension (shape, distances, neighbourhood, topologies, etc.) is crucial to OBIA methods, and that this is a major reason for the remarkable increase in the use of segmentation-based methods in recent times, compared to the use of image segmentation in remote sensing during the 1980s and 1990s (Benz *et al.* 2004, Blaschke *et al.* 2014). Still, we can conclude that in very recent literature, segmentation has been seen as less crucial for OBIA. Authors increasingly recognise that the real potential lies in the intelligence and the chance to formulate user knowledge as ‘rule sets’ (Hofmann *et al.* 2011, Belgiu *et al.* 2014a, Lang *et al.* 2014).

Although various studies in literature report an increase in spatial and thematic accuracies for OBIA approaches (see the meta-analyses of Blaschke (2010) and Blaschke *et al.* (2014)), the creation of robust, object-based solutions for automated image analysis of a set of images or even large image archives still remains extremely challenging (Pinz 2005, Walker and Blaschke 2008, Hofmann *et al.* 2011, Laliberte and Rango 2011, Kohli *et al.* 2013). Especially the highly complex content of VHR image data and the hardly predictable variability of the objects’ qualities in such diverging image data reduce the robustness and transferability of OBIA rule sets used for classification. Consequently, either the rule sets or the objects’ shape or even both need manual adaptation in order to achieve acceptable results. However, manual interaction and adaptation is deemed to be time consuming, labour-intensive and consequently error-prone. Novack *et al.* (2014) report on results from transferring a generic knowledge base to two different software packages for OBIA, both of them finally operating with software-specific rule sets. In order to overcome the limited transferability of OBIA rule sets, we investigate the coupling, extension and integration of OBIA with principles and methods from the agent-based paradigm. In particular, this article introduces a framework for agent-based image analysis (ABIA), which extends the existing OBIA concepts and methods by some from the agent-based paradigm.

### 1.1. The principles of OBIA workflow

Recently, the typical workflow of OBIA begins with a more or less arbitrary segmentation of the input data to generate a hierarchical net of image objects followed by an initial classification of the generated image objects. Then, OBIA enters an iterative process of selective segmentation improvements and re-classifications until a satisfactory result is achieved. The criteria on which the subset selection has to operate during iteration can be based on spatial criteria, non-spatial criteria or both (Baatz *et al.* 2008, Lang 2008). In order to be able to reapply the segmentation and classification process, all processing steps, their procedural sequences and sub-sequences are organised in a rule set. Depending on the software used, rule sets can be described in a domain-specific language (DSL), which is structured as a programming language but uses linguistic elements and concepts of the application domain (Hudak 1996, Fowler 2010). A prominent representative of such a DSL in the context of OBIA is the cognition network language (CNL), which is implemented in the software eCognition® (Athelogou *et al.* 2007). In CNL, two principal types of rules exist: processing rules, which either calculate values or change the objects’ shape; and classification rules, which assign objects to classes based on defined classification rules. For the latter, hierarchical fuzzy classification schemes complement the usual threshold-based classification and thereby describe each class based on fuzzy membership functions and their combination to fuzzy rules (Benz *et al.* 2004). The hierarchical classification schemes reflect the object classes’ ontology, which describes the appearance of the real-world objects in the image data at hand (Belgiu *et al.* 2014b). However, the effort to develop a rule set can be great (Arvor *et al.* 2013, Belgiu *et al.* 2014b), and its reusability is limited once the input data changes (Hofmann *et al.* 2011). In the remainder of this article we therefore introduce a first approximation of a solution to this problem, which aims to integrate concepts of agent-based computing for the adaption of such rule sets and image objects.

### 1.2. The definition of quality in OBIA

Quality of geographic information and therefore of results derived from the analysis of remote-sensing data by definition reflects the minimum properties of an acceptable classification result (ISO 19157:^2013^). In this context, quality is usually determined by quantifying the correctness and completeness of a classification result. In remote-sensing practice, both aspects are measured by comparing a to-be-evaluated classification result with another classification result that is assumed to be true (Congalton and Green 2009, ISO 191577:^2013^, Novack *et al.* 2014). However, this approach suggests an absolute correctness and completeness of the reference classification that is not necessarily given. Additionally, it requires semantically absolute conform class definitions, which in many cases do not exist (Hofmann and Lohmann 2007, Albrecht *et al.* 2010). Hence, for evaluating the quality of a classification result, it is rather sensible to focus on the requirements a classification result has to meet in order to be accepted by the user. Such quality criteria could be, for example, a minimum allowed deviation from a given – and not necessarily correct – reference map. When using fuzzy classification mechanisms, further requirements can be defined; for example, a minimum allowed ambiguity per object, per class or per scene (Benz *et al.* 2004, Hofmann *et al.* 2011).

### 1.3. Software agents and multi-agent systems

**Figure 1.**
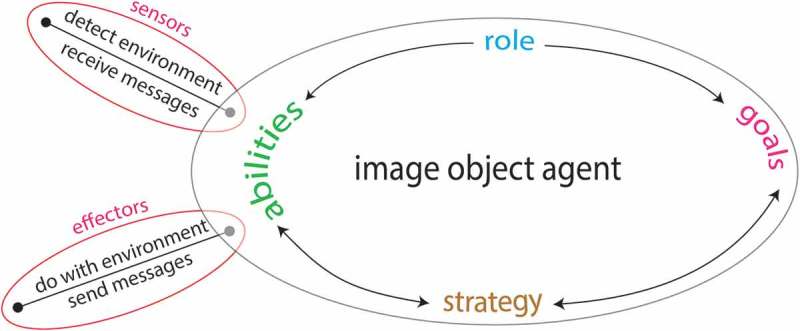
Principal components of a software agent.

Software agents are defined by being flexible and capable of acting autonomously in complex environments. They are thus provided with sensors and effectors in order to interact with their environment and to achieve predefined goals. Coupling several software agents to a multi-agent system (MAS) enables them to interact, communicate and collaborate among themselves in order to achieve either individual or common goals. In such a system, individual agents with different roles can exist, with each of them having role-dependent abilities and goals and all of them being organised in a network of collaborative agents. Although being organised in a network of software agents, each agent decides individually and based on its own strategy, how to act in a particular situation (Figure 1). These fundamental abilities have been shown to allow agent-based systems to deal with complex and unpredictable situations, as well as with incomplete information in a much more flexible and robust manner compared to conventional systems (Wooldridge and Jennings 1995, Jennings 2000).

#### 1.3.1. Software agents and MAS in GIS and image analysis

In GIScience, agent-based models (ABMs) are typically used for simulating complex spatiotemporal processes, such as land-use modelling (Parker *et al.* 2003, Macal and North 2005, Brown *et al.* 2005, Koch 2007, Marceau and Moreno 2008, Yu and Peuquet 2009). Likewise, agent-based systems (ABSs) are meanwhile widely used in industry for applications, such as process automation, that require a high level of robustness and flexibility (Fazel Zarandi and Ahmadpour 2009, Göhner 2013). However, using software agents or MAS in image analysis is not very common yet. In the remote-sensing domain, Samadzadegan *et al.* (2009) and (2010) report on the application of ABS for building detection from LiDAR data, whereas for image analysis in the life science domain, Bovenkamp *et al.* (2004) describe a similar approach for object detection in intra-vascular ultra-sound (IVUS) images and Rodin *et al.* (2004) demonstrate the application of agent-based systems in biological image analysis. Mahmoudi *et al.* (2013) describe an ABS designed to improve OBIA classification results in urban areas. Here, different tasks of object recognition are distributed among respectively defined agents. The agents then simultaneously operate on the image and share their (intermediate) results. The authors demonstrate that software agents can principally be used to parallelise image processing tasks and show how simultaneously arising individual classification results can be improved and condensed by agent-based systems as compared to conventional classification techniques.

#### 1.3.2. Ontologies in GIS, OBIA and the agent-based paradigm

Ontologies in GIS, OBIA and agent-based programming play a central role in the design of geo-databases, rule sets and that of software agents. In all the three domains, they explicitly describe those parts of the real world that are relevant for the respective domain and application. Especially in OBIA, the ontology acts as the foundation for the rule set, its object classes and all the classes’ semantic constraints. It describes the object classes and their semantics as independent from any underlying image data as possible. However, the image-specific appearance of particular classes in particular image data can only be described by a rule set that uses the structure of the ontology but is simultaneously designed for the data in use. Belgiu *et al*. (2014b) describe a method for converting an ontology expressed in the Web Ontology Language 2 (OWL 2, see Motik *et al.* 2009) automatically to a framework for an OBIA rule set. OWL 2 is a recommendation of the World Wide Web Consortium (W3C) that has been widely adopted by the Semantic Web Initiative. The remote-sensing ontologies extend the semantics of target classes such as land cover classes, informal settlements or refugee camps with properties that allow their detection in the remotely sensed data at hand. These properties are acquired either from literature (Belgiu *et al.* 2014c) or by using data mining techniques (Belgiu *et al.* 2014a, Maillot *et al.* 2004). In agent-based computing for the design of software agents, the so-called belief-desire-intention (BDI) model is very common (Rao and Georgeff 1995). In this context the ontology serves as the world-model for agents. That is, the ontology describes the agents’ beliefs. As recent studies showed (Viezzer 2006), this world model is not necessarily static. Moreover, software agents can be designed to adapt their world-model according to changing environmental conditions as a kind of an individual survival strategy.

## 2. Integrating OBIA and the agent-based paradigm

The concept for ABIA proposed in this article focuses on integrating mechanisms of agent-based control (ABC) as applied in process automation and ABM with OBIA. In particular, a framework for autonomous and adaptive control of OBIA using software agents in order to increase the robustness of particular OBIA solutions is introduced. For this purpose, two principal and independent approaches in the MAS framework are possible:

1. Extending the image object hierarchy as known from OBIA to a hierarchically organised MAS of networked image object agents (IOAs) with autonomous adaptation and interaction capabilities.
2. Autonomously adapting existing rule sets by means of a MAS of rule set adaptation agents (RSAAs) in order to robustly analyse varying image data without any need for further human interaction.

**Figure 2.**
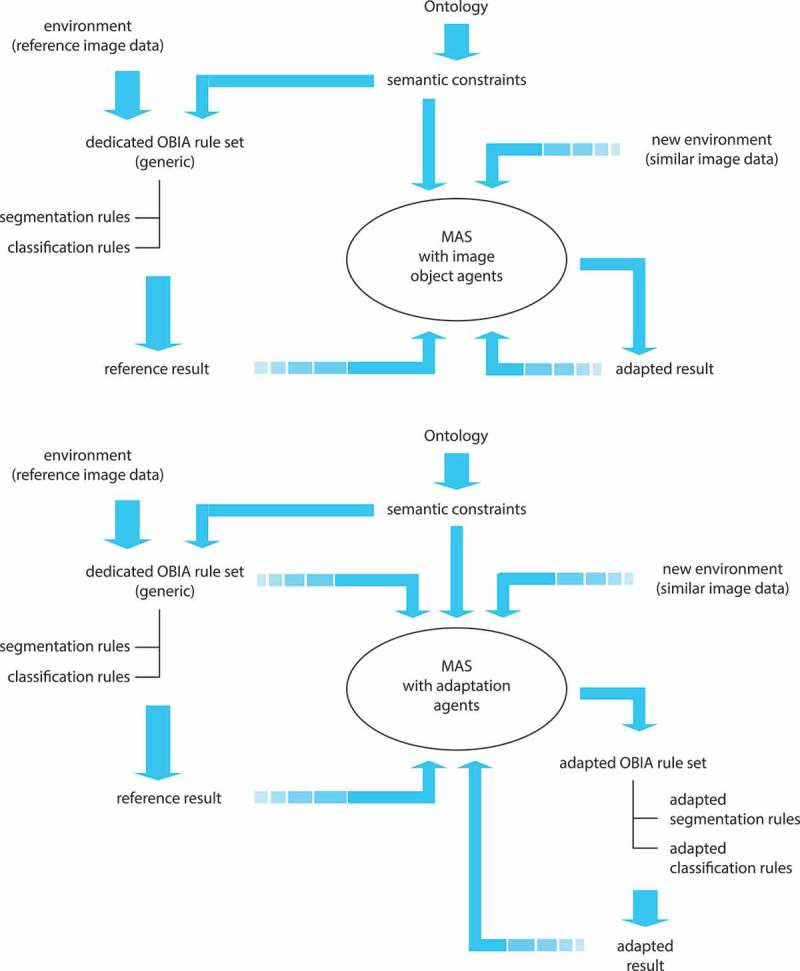
Principal workflow for ABIA with MAS operating with RSAAs (top) and IOAs (bottom).

In the following we focus on the first approach. In this approach, we extend the well-known OBIA concept of a hierarchical net of image objects to a hierarchical net of IOAs. Within this network, each image object aka image segment can act and interact in agent-based manner, that is, act and react, cooperate and negotiate with other agents in order to achieve its goals (Figure 2).

### 2.1. Conceptual framework

In the context of ABIA, each MAS must balance its activities between ontology conformity and quality requirements. Consequently, in the course of adaptation, each MAS must detect to what degree the quality requirements are met by particular adaptations and autonomously decide whether the adaptation process needs to be continued or not. By logging the success of each adaptation action, ‘promising’ adaptation strategies can be learnt or even the agent’s ontology can be adapted. That is, particular actions – preferably those providing the least ontology violation together with best achievable quality – are being prioritised for future applications. In the ABIA framework, this balancing and evaluation task can be either performed on an agent individual level, or by one or several dedicated control agents (CAs). The major roles of CAs are then:

1. Avoiding contradictions between the ontology and intended agent actions.
2. Compare the achieved quality with the user-defined quality for acceptance.
3. Trigger and abort adaptation processes as necessary; for example, abort if quality criteria are achieved.
4. Learn the most promising strategies for goal achievement.

### 2.2. Design of a MAS with IOAs

**Figure 3.**
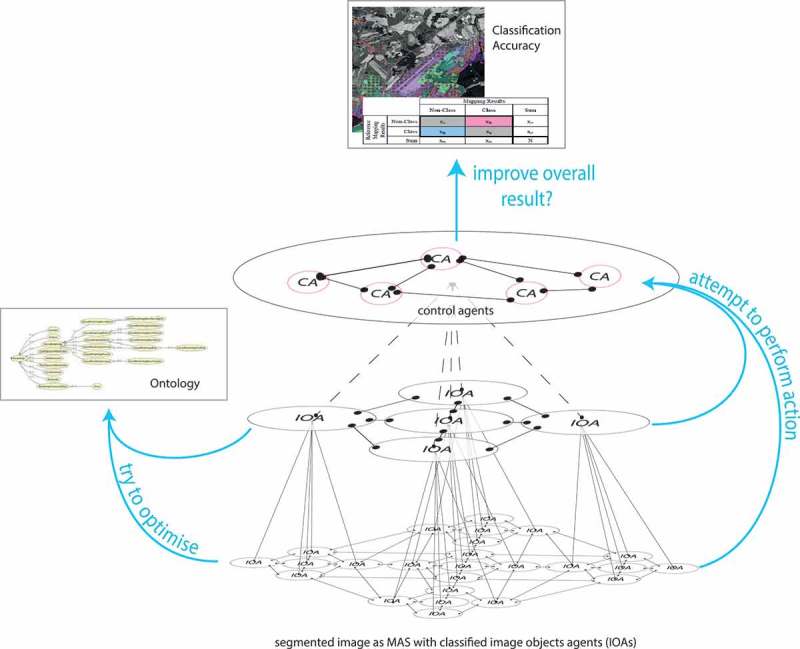
Hierarchical net of IOAs after segmentation and classification.

In a MAS consisting of IOAs, it is the image objects aka IOAs themselves that adapt autonomously according to changing image data. Similar to OBIA, in this architecture, image objects evolve to a hierarchical net of IOAs wherein each IOA is connected with its neighbour agents, its higher-level (super-) agents and its lower-level (sub-) agents (Figure 3).

**Figure 4.**
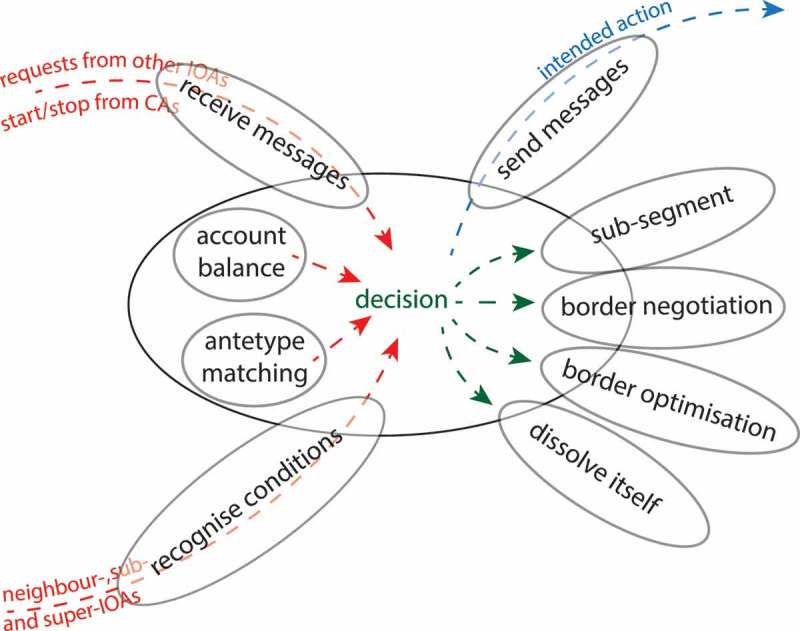
Architecture of an IOA with sensors to detect its neighbourhood and to receive messages from other agents. Its effectors allow the IOA to send messages and to change its own shape including dissolving itself.

After initial segmentation and classification steps, each IOA compares its degree of compliance with the ‘antetype’ of the class it was initially assigned to and as it is defined in the ontology. The goal of each IOA is to meet its ‘antetype’ as best as possible. For this purpose, each IOA develops its individual strategy to achieve this goal, whereas action priorities can be pre-defined in general or depending on the initial class assignment of an IOA. In principle, each IOA has two options to act: (1) re-segment itself and (2) merge with neighbouring IOAs. Option (2) is sensible in situations where merging of neighbour-IOAs would improve the overall classification quality (over-segmentation), but it implies that at least one of the involved IOAs will dissolve itself. Option (1) can be manifold, ranging from sub-segmentation via shrink-and-grow methods to negotiations about border pixels with neighbour IOAs. The prioritisation of particular actions can depend on the IOA’s class assignment and its grade of goal achievement. For example, an IOA classified as a ‘house’ but with borders that are not fully straight-lined might intend to execute a respective straight-lining algorithm. Since these operations can lead to conflicts between neighbouring objects (for example, between a ‘house’ and a ‘forest’ IOA), using CAs to prioritise individual actions is sensible (Figure 4).

**Figure 5.**
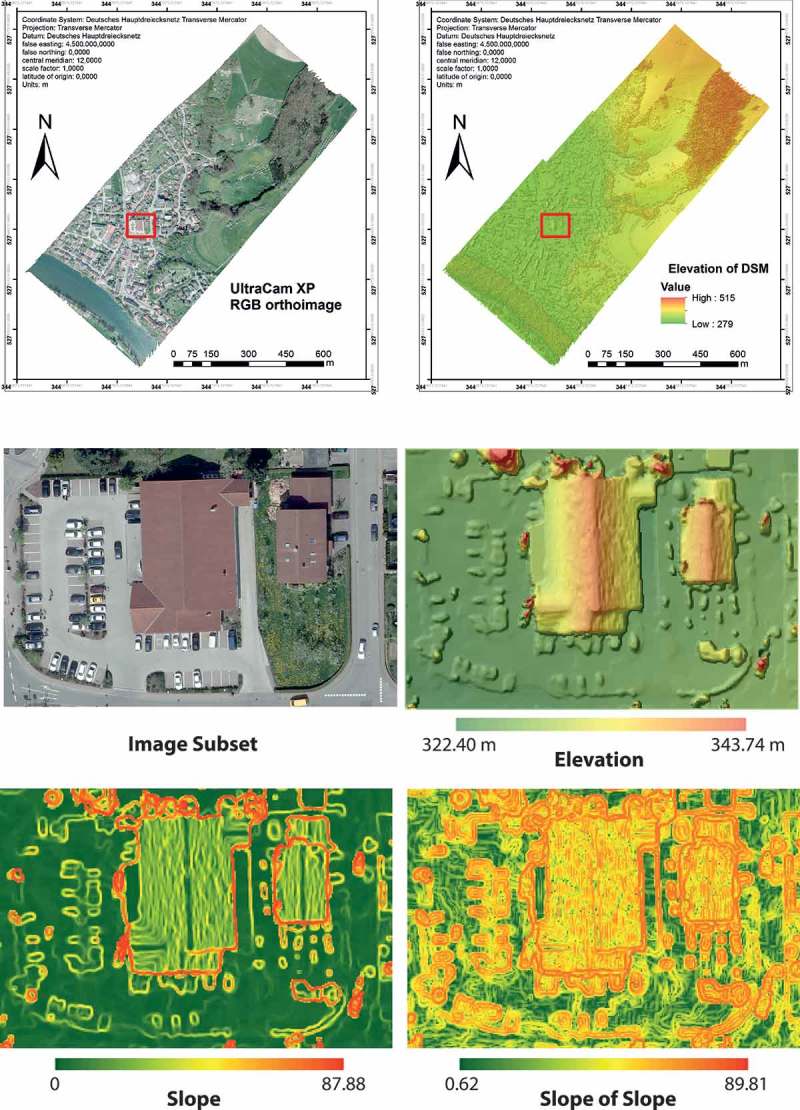
Orthoimage and generated DSM; red rectangular indicates subset under investigation (top). Image data (middle left) and DSM superimposed to hill shaded relief (middle right); slope and slope of slope (bottom, both in degree) for subset area.

The adaptation process stops either if the user defined quality requirements are met or if a user-defined threshold for the total number of adaptations has been reached. In order to avoid the system being trapped in a cyclic sequence of adaptation steps, a snapshot mechanism is sensible, which avoids endless loops.

## 3. Preliminary results and discussions

Since we are just in the beginning of implementing the framework, the following results are based on a simulated MAS created with eCognition^©^ Developer 9 (www.ecognition.com) and its CNL. That is, as far as possible an agent environment and IOAs are created without the use of a dedicated design tool for software agents; for example, Repast (Macal and North 2005). Consequently, our implementation in CNL is relatively simple. We provide example results that we have achieved with the created IOA-architecture as described in Section 2.2. For this purpose, simulated software agents are improving an initially created segmentation and classification result in a VHR data subset. The initial rule set is based on a simple building ontology (see Sections 1.3.2 and 3.2) and contains all necessary processing steps together with the class descriptions. In the subset, three buildings are visible that cannot be delineated properly by the initial rule set (Figure 8). Therefore, the IOA-MAS intends to stepwise improve these results as much as possible.

### 3.1. Data

The simulation has been applied on an orthorectified image from Weilheim, Upper Bavaria, Germany, taken in May 2010. The orthoimage together with a digital surface model (DSM) was generated based on a stereo pair of the RGB-bands captured by an UltraCam XP (www.ultracamx.com) using software from SimActive (www.simactive.com). The spatial resolution of the orthoimage is at 8 cm, that of the DSM at 35 cm. The radiometric resolution of the optical data is at 8bit. From the DSM, the slope and slope of slope (change of slope) were calculated per pixel and expressed in degree from 0° to 90°. The subset under investigation has a size of 1311 × 869 pixels (Figure 5).

### 3.2. Ontology

**Figure 6.**
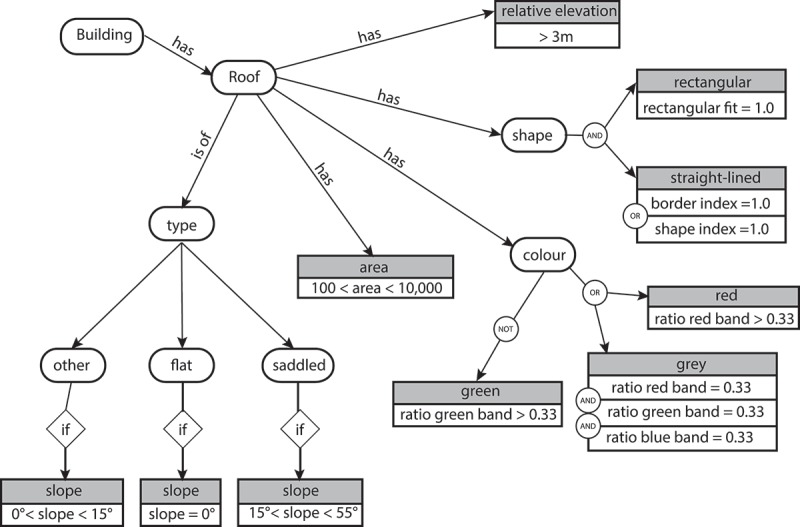
Ontology for building description in the used image and elevation data. Relations with numeric values are to be understood as fuzzy relations.

To describe semantically how buildings look like in general when using the above-described data, we have developed an ontology in accordance with Lampoltshammer and Heistracher (2014), Belgiu *et al.* (2014c) and Durand *et al.* (2007). In particular our ontology denotes the buildings’ roof shape, that is, their form and colour and the buildings’ relative height to their neighbouring objects. In contrast to the above-mentioned authors, in our building ontology colour and local elevation difference together with the elevation variability is included (Figure 6).

The ontology has been directly implemented as a respective class hierarchy in eCognition^©^ Developer 9 (see Table 1).

**Table 1.** Fuzzy classification rules for ‘roof’.

Parent class	Class	Fuzzy operator			Property	Membership function	Lower bound	Upper bound
n.a.	Roof	and (min)			Area		10 m^2^	100 m^2^
					Area		10,000 m^2^	15,000 m^2^
					Mean Diff. to neighbours DSM (0)		0.0 m	3.5 m
					Mean slope of slope		88°	90°
					Ratio green		0.32	0.34
			or (max)		Ratio red		0.28	0.33
				and (min)	Ratio blue		0.31	0.35
					Ratio green		0.31	0.35
					Ratio red		0.31	0.35
			or (max)		Border index		1.2	3.0
					Shape index		1.3	3.0
					Rectangular fit		0.5	1.0
Roof	Flat roof	and (min)			Mean slope		5°	10°
					Mean slope of slope		60°	80°
					Standard deviation slope		0.0	4.0
Roof	Saddled roof	and (min)			Mean slope		10°	60°
					Mean slope of slope		80°	85°
Roof	Other roof	and (min)		not	Flat roof	n.a	n.a	n.a
				not	Saddled roof	n.a.	n.a.	n.a.

### 3.3. Initial rule set

For initial classification, we have developed a simple rule set to delineate roofs based on the ontology as described in Section 3.2. It starts with a multi-resolution segmentation (MRS) as described by Baatz and Schäpe (2000) whereas all three bands, the DSM and the slope have been weighted equally for the segmentation. Respectively, the homogeneity criteria of the MRS are composed equally of the RGB bands, the DSM and the slope. The brightness per object is calculated only based on the RGB-bands. To assign the initially created objects to a ‘roof’ class, a fuzzy class hierarchy has been defined with classification rules as depicted in Table 1. Applying the above-described rule set to the data leads to an initial classification result as depicted in Figure 8.

### 3.4. Simulation of an IOA-MAS

In order to simulate a MAS consisting of IOAs, image objects are virtually organised as IOAs in eCognition^©^. In our particular case, each object aka IOA having a membership degree of µ > 0.0 to one of the ‘roof’ classes intends to become a best member of its original class or one of the other ‘roof’ classes (goal). To achieve this goal, each IOA is provided in principle with all eCognition^©^ operations an object can perform. However, for the sake of convenience in our example, each IOA is just provided with the following effectors:

1. merge with most promising neighbour object;
2. coat for five pixels;
3. do nothing.

For achieving its goal, each IOA can decide at every processing step to apply one of these effectors without any priority. During processing, to decide which of them seems to be the most promising, they are executed virtually for each object-agent on a copy of the current scene (**evaluate** in Listing 1; see supplement for a detailed description of the CNL code). We have disclaimed to define further quality criteria – such as a minimum number of objects with acceptable quality – as common goals. Consequently, no CAs were implemented. That is, the overall processing stops after a user-defined number of steps.^1^ Since in eCognition^©^ all objects are embedded in a (hierarchical) net of image objects, a neighbourhood sensor for each IOA is not necessary. The inner status of each IOA (goal achievement) is expressed by the current membership degree µ to the best-fitting ‘roof’ class, which is determined during processing by classifying each object after applying one of the effectors. Since for merging at least one of the merging objects has to abandon itself, this is only allowed if the resulting object has a better membership than both objects had before merging. This way, negotiation (function *IsAllowdMerge()* in Listing 1) between neighbouring IAOs is simulated (see Listing 1 and supplement for detailed description).

Listing 1: Pseudo-code to describe the behaviour of IOAs being classified as one of the ‘roof’-classes.


					**ImageObjectAgent**
					*roof*
				


					**goal**
					*MemberOfClassRoof*
				

    µ(*roof*, **this**) = 1.0;
				


					**effector**
					*Merge*
				

    merge(**this**, NeighbourObject);
				


					**effector**
					*CoatFivePixels*
				

    coat(**this**, 5 Pixels, *<default eCognition params>*);
				


					**effector**
					*DoNothing*
				

   *…*
				


					**sensor**
					*MyNeighbours*
				

    ListOfNeighbours(*<from eCognition>*)
				


					**evaluate**
					*BestMerge*
				

    **for_all** NeighbourObjects
				

          **if** IsMax(µ(*roof*, merge(**this**, CurrentNeighbour)) **and**
				


					*          IsAllowedMerge*(**this**, CurrentNeighbor) **then**
				

          merge(**this**, CurrentNeighbour);
				

          merge_µ = µ(*roof*, merge(**this**, CurrentNeighbour));
				

          **endif**
				

    **endfor**
				


					**evaluate**
					*Coating*
				


					*    CoatFivePixels*;
				

    coat_µ = µ(*roof*, **this**);
				


					**if** merge_µ > coat_µ **then**
					*Merge*
					**elseif** coat_µ > current_µ **then**
					*CoatFivePixels*
					**endif**
				


					**endif**
				

### 3.5. Results and discussion

**Figure 7.**
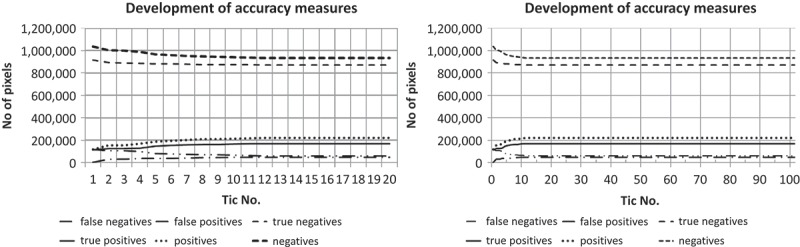
Development of accuracy measures during processing for 0–17 tics (left) and 0–100 tics (right).

**Figure 8.**
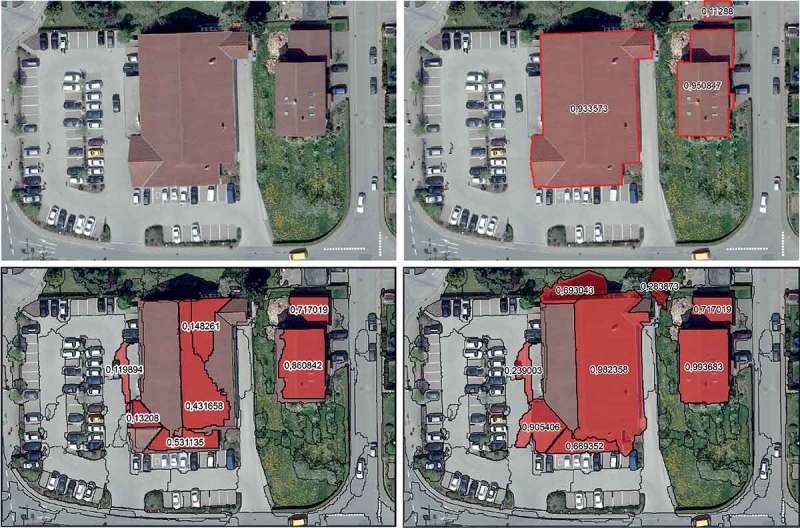
Subset image data (top left), manual reference (top right), initial (bottom left) and final (bottom right) result after ABIA processing, with membership degrees to ‘roof’ per object.

Applying the simulated IOA-MAS as described in the section before to the initial result generated with the data and rule set described in Sections 3.1 and 3.3 led to the final result as displayed in Figure 8. We have run the ABIA process for 100 tics. In order to document the development of the classification quality, after each tic the intermediate result (see Appendix) underwent a per-pixel accuracy assessment based on correctly assigned pixels (true positives and negatives) and wrongly assigned pixels (false positives and negatives) using the manual reference image as depicted in Figure 8. Additionally, some quality assessment measures based on the membership degree of each object are calculated for each intermediate result. As can be seen from the development plots, after 17 tics no significant changes are observable (Figure 7). Thus, in the following discussion we are focusing on the first 17 tics.

Comparing the final result with the initial result and the reference map visually, the following can be observed: The over-segmentation of the big building at the centre has been reduced although the left part still remains unclassified (the default threshold for crisp class assignment in eCognition^©^ is at µ ≥ 0.1). The initially over-segmented and not fully outlined building in the central-east has improved to an almost perfectly represented ‘roof’ while the belonging garage did not change at all and was not merged with the main building, as in the reference map. The shaded vegetation close to the central building in-between the buildings has been wrongly outlined and classified as ‘roof’ after the ABIA process. Additionally, objects being initially wrongly classified as ‘roof’ at the border between the parking lot and the central building grew or even merged with the building segments, while the western part of the big central building’s roof remained unclassified (but it could improve its membership to ‘roof’ from 0.0 to 0.016; see Appendix). The unclassified cut-off part of the garage in the north-east still remained unclassified and never changed during the process. Note that its membership degree to ‘roof’ even in the reference map is relatively low (Figure 8).

**Figure 9.**
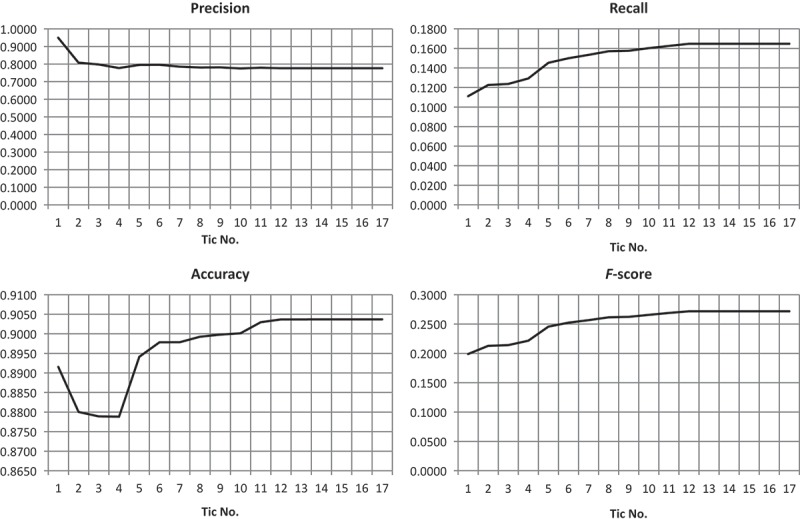
Development of precision, recall, accuracy and F-score during the ABIA process.

Based on the basic per-pixel accuracy measures, we have calculated for the first 17 intermediate results the derivative measures: precision, recall, accuracy (Landgrebe *et al.* 2006) and F-score (Powers 2011). Additionally, we have tracked the average membership degree of all objects with a membership to ‘roof’ of *µ* > 0.0 and the number of objects with µ > 0.5. While the former documents the average adaptation of the IOAs, the latter documents the classification’s fuzziness on a per object basis (Siler and Buckley 2005). Further, we have observed on a per object basis the average membership to ‘roof’ of those objects, which cover at least 50% true positive pixels (predominantly correct objects). Similarly, we have observed the average membership to ‘roof’ of predominantly false positive objects, that is, of objects that cover more than 50% false positive pixels. While the former documents the improvement of objects, the latter documents the development of false positive errors (Figure 9).

**Figure 10.**
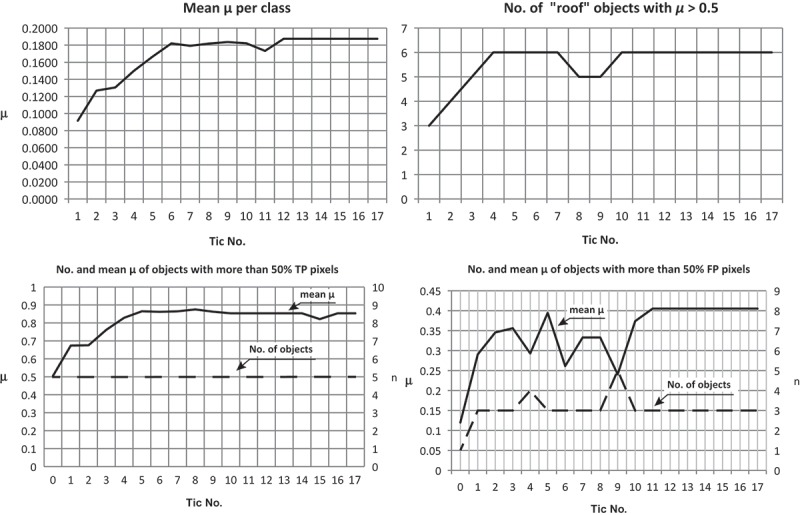
Development of accuracies based on membership degrees per object. Mean membership to ‘roof’ (upper left), number of objects with a membership degree of *µ* > 0.5 to ‘roof’ (upper right), mean membership degree of objects with more than 50% true positive pixels (bottom left) and more than 50% false positive pixels (bottom right).

As can be seen from Figure 9, the accuracy first decreases (tic No. 1–4), but then increases until it saturates at tic No. 12 at a level of 0.9037. Similarly, recall even continuously increases already from the very beginning, while precision more or less inversely decreases in the same period of tics; the F-score behaves similarly to recall. This indicates that the overall classification has increased during the ABIA process. Regarding the classification quality based on the membership degrees per object, this has improved as well (Figure 10).

The mean µ of all objects with a membership to ‘roof’ of µ > 0.0 (mean µ per class) increases from 0.0916 to its final saturated level of 0.1874, whereas the mean µ per class also includes wrongly assigned objects (false positives) and objects with a relatively low membership degree. The number of ‘roof’ objects with µ > 0.5 develops from *n* = 3 (tic No. 1) to *n* = 6 (tic No. 10) whereas in tic No. 8 and tic No. 9 it decreases to *n* = 5 but then returns to *n* = 6. This indicates that the number of classified ‘roof’-objects with low fuzziness, that is, with a relatively clear class assignment, has increased.

Comparing the developments with the reference classification, obviously a per-object improvement is observable, since the number of objects with a minimum amount of 50% correctly classified pixels remained constant (*n* = 5) but their average membership degree to ‘roof’ has increased and remains at a relative high level (mean µ = 0.85). Vice versa, the initial number of wrongly assigned objects (more than 50% of false positive pixels) increases and they could improve their average membership degree, too, but on a far lower level (mean µ = 0.40) than the correctly assigned objects. Similarly, the obviously largest false negative object, that is, the western part of the big central building, develops positive (µ = 0.0 in tic No. 1 to µ = 0.016 in tic No. 11 and the following tics). However, this object could not improve further, because if the eastern ‘roof’ object of the centre building (µ = 0.98 in tic No. 17) would merge with it, the resulting object would have a decreased membership to ‘roof’. Similarly, a growth of this object would decrease the membership of the neighbouring ‘roof’ objects. Moreover, when comparing the memberships of the final result with those of the reference map (Figure 8), the manually classified objects (except the cut-off object in the north-east) have lower membership degrees to ‘roof’ than some of the automatically processed ‘roofs’. Especially for the building in the centre-east this is obvious. This indicates, that (1) the ontology and therefore the class hierarchy is not absolutely in compliance with our perception and (2) data quality leads to misinterpretations by the classifier. The latter is obvious for shaded higher vegetation, since here elevation information and spectral information for vegetation and buildings are similar due to lower brightness. Especially in the case of the shaded vegetation bordering north to the centre building (µ = 0.69 in tic No. 17), it has increased its membership to ‘roof’, since the shade of the bordering building improves its shape in terms of being ‘roof’-like. For this object during the ABIA process, the more shade (of the building) it has accumulated the more its shape criteria for ‘roof’ was fulfilled. Nevertheless, for all false positive objects including both wrongly assigned vegetation objects, their membership degree to ‘roof’ is relatively low. Additionally, when examining Figure 5, at some positions, simply the DSM is very inaccurate. Especially at the buildings’ borders, this effect led to a slight overgrowing of, and in the worst case to a merger with, the already wrongly assigned ‘roof’ objects, as is the case in the south-eastern jutty of the central building.

## 4. Conclusions and outlook

The article introduces a framework for agent-based image analysis of remote-sensing data in order to overcome the problems arising with robustness of OBIA rule sets and their adaptability to a variety of similar images. In particular, it suggests two principal possible conceptual approaches of integrating OBIA with methods from agent-based control and simulation (IOAs vs. RSAAs) while it is focusing on the IOA-approach.

A rule set has been implemented in CNL and applied with eCognition^©^ Developer 9, which initially detects buildings quite fairly in a small subset of an UltraCamXP orthoimage and a DSL based on these data. The rule set further simulates the behaviour of relatively simple designed IOAs, which stepwise improves their initial results.

The agent-based processing and its interim-results have been analysed under the aspects of: per-pixel accuracy improvement and goal achievement. Although the final results still show deficits, an improvement of the initial result could be observed. Some classification deficits are obviously data driven. The latter could be improved for example by including infrared information. Introducing brightness as a further colour criterion for the building-ontology and rule set would be another option, whereas this adaptation should be realised either by the IOAs themselves or by RSAAs. The design of the presented IOAs is certainly very simple, which is to some extent due to the limits of eCognition^©^ and CNL. Both are not intended to be used for agent-based programming and consequently the typical BDI paradigm for software agents could only be implemented slightly, that is, a MAS consisting of IOAs could only be simulated. However, it is planned in future work to use more dedicated development environments, such as Repast. Then, for example IOAs could be enabled to recognise which of the class describing features cause a low ‘roof’ membership and based on that, trigger a more dedicated self-improving operation.

The fact that objects of the manual reference did not fully fulfil the membership criteria of ‘roof’ indicates that the building ontology used and the perception applied for reference generation are diverging. Thus, the ontology should be further developed in order to meet more aspects of ‘roofs’, which could then improve the fuzzy classification rules.

Nevertheless, we have shown that even with these simply designed IOAs, an improvement of the initial classification is possible. We have analysed and presented these improvements under the aspects of compliance with the reference map and reliability of fuzzy class assignments. The potential of ABIA to autonomously adapt image objects to unknown imaging situations has been demonstrated.

## Disclosure statement

No potential conflict of interest was reported by the authors.

## Supplemental data

Supplemental data for this article can be accessed here.

Supplemental data: description and instructions

Video capture of the agent-based analysis process together with the development of accuracy measures

eCognition ruleset containing the whole simulated agent-based analysis process

Raster data (UltraCam RGB, DSM, slope and slope of slope) used for image analysis and accuracy assessment (reference) in eCognition

## Note

1. In agent-based modelling these steps are called tics. Each tic represents one elapsed epoch of the model.
